# Extirpation of the Uterus

**Published:** 1880-02

**Authors:** 


EXTIRPATION OF THE UTERUS.—In a recent operative clinic in the Mercy Hospital, Prof. E. W. Jenks, Professor of Gynæcology in the Chicago Medical College, found it necessary to remove the *entire uterus* on account of malignant disease. Of course it involved a free opening through the pelvis into the abdominal cavity, and a temporary escape of the intestines. These were carefully replaced, and the operation finished by closing the vaginal opening with silver sutures. The patient sustained the operation well, and subsequently made a rapid and complete recovery. She left the hospital a few days since for her home, able to walk about freely.

